# “Delabeling” by direct provocation testing in children and adolescents with a suspected history of a delayed reaction to β-lactam antibiotics: Consensus paper of Gesellschaft für pädiatrische Allergologie und Umweltmedizin (GPAU), Deutsche Gesellschaft für Allergologie und klinische Immunologie (DGAKI), and Ärzteverband deutscher Allergologen (ÄDA)

**DOI:** 10.5414/ALX02480E

**Published:** 2024-05-31

**Authors:** Irena Neustädter, Sophie Blatt, Gerda Wurpts, Heinrich Dickel, Christian Walter, Werner Aberer, Sebastian Bode, Timo Buhl, Sunhild Gernert, Susanne Harner, Guido Heine, Sebastian Kerzel, Meike Köhler, Lars Lange, Joachim List, Hans F. Merk, Thomas Nüßlein, Hagen Ott, Franziska Sattler, Antje Schuster, Helen Straube, Bettina Wedi, Torsten Zuberbier, Knut Brockow

**Affiliations:** 1 Pediatric and Adolescent Medicine, Diakoneo Klinik Hallerwiese-Cnopfsche Kinderklinik, Nuremberg,; 2 Clinic and Polyclinic for Dermatology and Allergology at Biederstein, Technical University of Munich, Munich,; 3 Clinic for Dermatology and Allergology, Aachen Comprehensive Allergy Center (ACAC), University Hospital of RWTH Aachen University, Aachen,; 4 Clinic for Dermatology, Venereology and Allergology, St. Josef Hospital, University Hospital of the Ruhr University Bochum, Bochum,; 5 Practice for Pediatric and Adolescent Medicine, Allergology, Bad Homburg, Germany,; 6 Department of Dermatology and Venereology, Medical University of Graz, Austria,; 7 University Clinic for Children and Adolescents, Ulm,; 8 Department of Dermatology, Venereology and Allergology, University Medical Center Göttingen,; 9 Department of Pediatrics, St. Marien Hospital, GFO Clinics, Bonn,; 10 Clinic and Polyclinic for Pediatrics and Adolescent Medicine, University of Regensburg, Regensburg,; 11 Department of Dermatology, Venereology and Allergology, University Medical Center Schleswig-Holstein, Campus Kiel, Kiel,; 12 Asthma and Allergy Outpatient Clinic, Dr. von Hauner Children’s Hospital, LMU University Hospital, Munich,; 13 University of Freiburg, Center for Pediatric and Adolescent Medicine, Freiburg,; 14 Clinic for Pediatrics and Adolescent Medicine, Gemeinschaftsklinikum Mittelrhein, Koblenz,; 15 Children’s and Youth Hospital Auf der Bult, Hanover,; 16 Clinic for General Pediatrics, Neonatology and Pediatric Cardiology, University Hospital Düsseldorf, Düsseldorf,; 17 Princess Margaret Children’s Hospital, Darmstadt,; 18 Hannover Medical School, Clinic for Dermatology, Allergology and Venereology, Hanover, and; 19 Allergology and Immunology, Department of Dermatology, Venereology and Allergology, Charité-Universitätsmedizin Berlin, Berlin, Germany

**Keywords:** allergy, β-lactams, benign rash, direct drug provocation testing

## Abstract

Background: Approximately 10% of European children are classified as allergic to drugs. In the majority of these children, no allergy to β-lactam antibiotics (BLA) can be found. In most cases, the exanthema is caused by the infection. Materials and methods: The objective of this paper is to describe the causes and consequences of a misdiagnosis of drug allergy. We propose a method for establishing a correct diagnosis in the case of a history of a delayed reaction during treatment with a BLA. For this purpose, a proposal was discussed via e-mail communication, and consensus was reached among the members of the drug allergy working groups of the participating medical societies. Results: The suspicion of a BLA allergy based on the medical history alone can have a negative impact on future antibiotic treatment. Exanthema associated with febrile infections not related to drug administration is a frequent finding in children. This makes it all the more important to be able to recommend a standardized procedure for clarification in children and adolescents with suspected hypersensitivity reactions. The medical history should be the basis on which to diagnose either a drug allergy or another possible differential diagnosis. A mild maculopapular exanthema (MPE) can be an expression of a drug allergy or a nonspecific viral exanthema. Uncomplicated MPE is not associated with significant systemic involvement, and there is no involvement of the mucous membranes or cutaneous blistering. Only a small number of children with uncomplicated MPE show positive skin tests and only ~ 7 – 16% of suspected BLA diagnoses can be confirmed by provocation tests. Thus, in children with uncomplicated MPE, drug provocation can be performed in an outpatient setting even without prior skin testing. This paper presents a 3-day outpatient direct provocation scheme for BLA delabeling in children with uncomplicated MPE. Conclusion: Many children and adolescents are unnecessarily denied treatment with BLA after an uncomplicated MPE while being treated with a BLA.

## Introduction 

β-lactam antibiotics (BLA) are the treatment of choice for many bacterial infections [2]. In children and adolescents, this group of drugs is also frequently associated with drug hypersensitivity reactions (off-target reactions) [4]. The overall incidence of suspected drug hypersensitivity reactions appears to be higher in children than in adults (10 – 12% versus ~ 5%) [9]. 

The occurrence of a cutaneous reaction at the same time as an illness treated with a BLA often leads to the suspicion of and labeling with BLA allergy – without subsequently verifying this suspicion with a valid diagnostic procedure. In children with febrile infections, infection-associated exanthema often occurs, which is not causally related to the drug administration, but is frequently misinterpreted as a drug allergy [4]. On the other hand, a mild maculopapular exanthema (MPE) can also be an expression of a drug allergy [4]. A BLA allergy can only be confirmed in a small number of patients after a diagnostic test (skin test and/or allergen-specific IgE) [4, 11]. An important tool is the medical history, which must include questions about the reaction interval, the clinical manifestation with a description of the morphological findings, in particular the skin reaction (photo documentation if necessary). 

**Note: On which day of treatment did the suspected allergic symptoms occur and what was the time interval from BLA administration?**

**MPE typically occurs 4 – 14 days after the start of therapy. In the case of a recuring reaction, the time interval is typically shorter compared to the initial reaction (in the case of MPE, a reaction is possible after just 6 hours or up to 4 days) [11].**

In addition, the recommendation to avoid β-lactams for the rest of the child’s life without adequate diagnostic confirmation represents a severe restriction of therapeutic options that lasts for decades and should be critically reviewed as part of the antimicrobial stewardship program [2]. 

## Characteristics of uncomplicated maculopapular exanthema or benign rash 

MPEs never affect the oral or anogenital mucous membranes, show no blistering or epidermolysis, and are only accompanied by mild to moderate pruritus without a deterioration in the child’s general condition. They usually resolve spontaneously and completely within a few days. It should be noted that parainfectious exanthema in children is often misinterpreted as a cutaneous drug reaction [13]. Due to the similarity between the symptoms in the early stages of viral and bacterial infections and/or because a bacterial superinfection is considered, many patients with viral infections receive antibiotics and antipyretics. Drug-induced T-cell stimulation can occur where there is a viral infection. This can cause an exanthema which without the above combination is unlikely to recur [9]. 

## Practical procedure 

The first step is therefore to check whether the clinical picture of the reaction is compatible with a drug allergy or whether an alternative diagnosis (e.g., viral exanthema) is more likely. Some patients may have been treated again with the suspected drug and have tolerated it since the suspected reaction. In these patients, the BLA allergy label can be removed without testing. This result must be explained in detail to those affected in order to avoid further avoidance of the antibiotic [11]. The check for “warning signs” as a contraindication for direct provocation testing is obligatory (Table 1) [12] 

Provocation tests are useful after clinically mild late reactions (uncomplicated MPE / benign rash), [3]. In children and adolescents, they are primarily used to rule out a drug allergy (delabeling) and have a very high negative predictive value. For late reactions, provocation tests could only confirm ~ 7 to max. 16% of suspected diagnoses [15]. 

## Provocation tests 

Sensitization of children and adolescents as an expression of a possible allergic reaction to a drug is very rare compared to that of adults [6]. To diagnose a delayed reaction, in the sense of an uncomplicated exanthema (MPE/benign rash), a drug provocation test can be carried out in children and adolescents without prior cutaneous testing [3, 5, 6, 12]. Studies have shown only a low sensitivity for cutaneous testing [15]. The significantly lower effort facilitates the delabeling of an assumed drug hypersensitivity [4]. 

It should be noted that in the case of severe late reactions such as epidermal necrolysis (EN) (including Steven-Johnson syndrome (SJS) / toxic epidermal necrolysis (TEN)), acute generalized exanthematous pustulosis (AGEP), drug reaction with eosinophilia and systemic symptoms (DRESS), and if warning signs are detected (Table 1), direct drug provocation testing should not be carried out. 

The patients and their caregivers are informed about the objectives and the reactions that may occur. It should be mentioned to them that the drug reaction usually corresponds to the index reaction. Skin testing is unnecessary in the case of late-type reactions with MPE. Delabeling can then be sought by means of direct oral provocation. It should also be made clear in advance that, should a drug reaction occur, an alternative appropriate drug must then be tested. 

The direct provocation scheme* presented in Table 2 is only indicated for safe uncomplicated MPE. 

[*Direct provocation means the performance of an oral provocation without prior cutaneous or serological testing after an appropriate medical history has been taken. The suspected drug is assigned to the patient after taking the medical history.] 

A single dose on day 1 followed by a break until the next day is safe and important (wash-out period [12]). On days 2 and 3, therapy can then be administered according to the recommended dosage in order to increase parental confidence in a provocation test where an allergic reaction does not occur [10, 12]. 

During the administration of provocation doses at home, any drug-induced reaction that occurs should be checked by a doctor. Additional photo documentation may be helpful. This should be considered when planning the provocation testing in order to ensure the best possible availability of the medical team. The timing of the provocation test should be planned to ensure that the medical team will be available. 

To ensure that the process of delabeling is accepted and successfully carried out by the parents, the children/adolescents should be free of infection at the time of provocation testing [9, 13]. 

The drug reactions that may occur generally correspond to the index reaction [6, 7] and can be controlled by antihistamines and, if necessary, glucocorticoids (prednisolone: 1 mg/kg/BW) [15]. 

**Figure 1:**
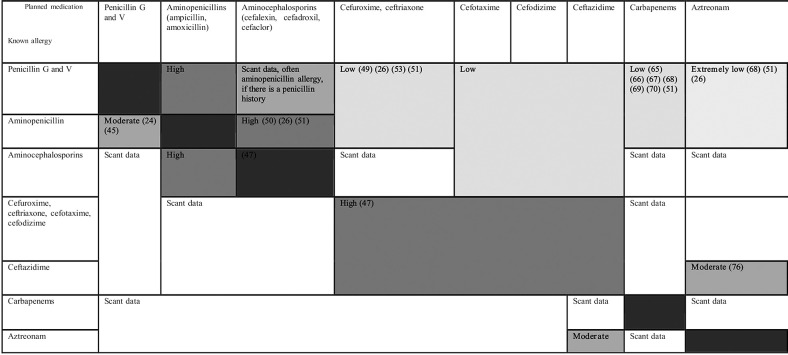
Estimates on the likelihood of allergic cross-reactions between the various β-lactam antibiotics ([14]).

If a BLA drug allergy is confirmed, an allergy certificate should be issued. It is recommended to check depending on the structural similarities and probabilities of allergic cross-reactions (Figure 1) whether drugs from the BLA group should be permitted or are contraindicated [14]. **If the suspicion of a drug allergy has not yet been confirmed, this should be documented as a suspected allergy until further provocation tests are carried out. The description should include the symptoms, the suspected trigger as well as the timeline of the reaction.**

The aim of clinicians must be to protect those affected from misdiagnosis and at the same time to safely diagnose drug allergy. It should be ensured that an updated allergy passport is issued and to find appropriate treatment alternatives if necessary. All measures should be discussed with all parties involved in a comprehensible manner. 

## Acknowledgment 

I would like to thank Gerda Wurpts, MD, and Prof. Knut Brockow, MD, for their critical review and support in the preparation of this article. 

We would also like to thank Dr. Deirdre Cahalane, Dublin, Ireland for her help and critical assistance with translating this text into English. 

## Authors’ contributions 

A proposal was discussed via e-mail communication, and consensus was reached among the members of the drug allergy working groups of the participating medical societies. Authorship: Neustädter I.; contributorship: other authors. 

## Funding 

No funding was received for this work by any of the authors. 

## Conflict of interest 

The author declared no potential conflict of interest with respect to the research, authorship, and/or publication of this article. 

**Table 1.** Warning signs of β-lactam allergy, modified according to [1, 12].

Immediate reaction	Delayed reaction
Conjunctival erythema	Pronounced facial edema
Shock symptoms (arterial hypotension/dizziness)	Atypical target lesions, bullous lesions
Coughing, sneezing, wheezing	Erythroderma
Dyspnea	Hemorrhagic and necrotic lesions
Hoarseness	Painful skin, mucous membrane involvement
Dysphagia	Generalized lymphadenopathy
	Pathological laboratory findings (e.g., liver enzyme elevation, impaired renal function)

**Table 2.** Provocation regime [6, 8, 15].

Day 1 (practice/clinic)	Day 2 (at home)	Day 3 (at home)
1 single dose	Daily dose distributed in 2 – 3 doses (depending on the antibiotic)	Daily dose distributed in 2 – 3 doses (depending on the antibiotic)
Monitoring for 1 hour	Start ~ 24 hours after the first dose day 1*	

*Wash-out period should be ~ 24 hours so that delayed reactions can be recorded after the 1^st^ dose during the time interval between reaching the therapeutic dose and the subsequent dose.
